# Personality differences between internal medicine and surgical residents in an Asian population

**DOI:** 10.1186/s12909-022-03689-w

**Published:** 2022-08-29

**Authors:** Lin Kyaw, Kep Yong Loh, Yi Quan Tan, Fiona Mei Wen Wu, Ho Yee Tiong, Ziting Wang

**Affiliations:** 1 Department of Urology, National University Health System, Singapore, Singapore; 2 Department of Internal Medicine, Singapore General Hospital, Singapore, Singapore

**Keywords:** Professional Development, Personal, Characteristics/Attitudes, Career Choice

## Abstract

**Purpose:**

Personality traits often have an impact on the way individuals relate to each other as colleagues and the patients we treat. It is often perceived that distinct personality exist between different specialties and may help predict success during one’s training and career.

**Methods:**

Objective of the study was to compare the personality between surgical and medical residents. Thirty-five medical residents and 35 surgical residents completed the Revised NEO Personality Inventory, a validated measure of personality traits. A score was generated for each of the 5 major character traits namely: neuroticism(N), extraversion(E), openness(O), conscientiousness(C), agreeableness(A). Each of these traits were subdivided into 6 component facets. This was compared with sociodemographic characteristics.

**Results:**

Medical residents displayed higher scores in the area of overall Agreeableness, with a mean score of 47.4 vs 40.5. Within Agreeableness facets, medical residents also displayed higher scores of straightforwardness, altruism and modesty. Surgical residents displayed higher scores in terms of overall Extraversion (52.4 vs 45.4). Within the Extraversion facets, surgical residents were also more assertive and excitement-seeking. There was no difference in the overall neuroticism domain; however, within the neuroticism facets, surgical residents had statistically higher mean scores in angry hostility and impulsiveness. Gender stratification did not result in any statistically significant difference.

**Conclusion:**

There are fundamental differences between personalities of medical and surgical residents. Detailed analysis of each individual’s data could be useful, with proper assistance and coaching, for residents in learning more about their personalities and how these impact their clinical practice. This can be beneficial in future career counselling and the development of a more holistic medical practitioner.

## Introduction

The utility of personality assessment is well recognised in multiple industries and occupations. Studies have demonstrated that personality inventories correlate various characteristics to higher-performing individuals working in high stress environments, such as aviation programmes and law-enforcement work [1, 2]. It is often perceived that distinct personality types may help predict success during the span of one’s career [3].

Within the arena of medical science, personality traits often have an impact on the way individuals relate to each other as colleagues as well as the patients they treat. Whilst a multitude of studies have reflected differences between each medical discipline, the traits of young residents-in-training have not been well elucidated [4]. Although previous research have studied the association between personality traits and future choice of specialisations among medical students [5–7], elucidating the traits of successful in-training residents may provide a better guide for resident selection programmes. The high attrition rates in residency as seen in the United States and United Kingdom further highlights the difficulties in trainee selection. The economic and social costs associated with these high dropout rates highlight a need to study factors related to such phenomena [8, 9].

As such, assessing variations in psychosocial and personality dimensions of medical and surgical trainees may aid in career counselling for medical students and resident selection for programmes utilising the Revised NEO Personality Inventory (NEO PI-R),^1^ a well validated measure of the five-factor model, we aim to provide a more holistic observation of the intrinsic differences between residents of different disciplines. Understanding the traits of thriving residents in the respective disciples will be useful in guiding the career choices of medical students and junior doctors. The objective of the study was to compare the personality traits between surgical residents and medical residents.

For context, medical education in Singapore is mainly a undergraduate medical curriculum (> 90%), with only a medical school offering post-graduate medical education. After graduating with a Bachelor of Medicine, Bachelor of Surgery (MBBS) or Doctor of Medicine (MD), new doctors undergo a compulsory 1 year of training (PGY1) before gaining full registration to practice. Candidates can only start applying for residency programmes in PGY1. As of the time of this paper, it is neither routine nor compulsory for residency programs to administer personality assessment inventories for their prospective residents during the selection period.

## Materials and methods

A total of 35 medical residents and 35 surgical residents from the National University Hospital System (NUHS) were invited to participate in the Revised NEO Personality Inventory, a validated measure of personality traits. Participants completed the self-report questionnaire at home or at a private workspace. Subjects were chosen based on simple random sampling, we obtained a list of residents from the residency program coordinators and assigned each resident a number. Following which, a random number generator was used and relevant residents were contacted to participate in the study. As residents were approached personally, the non-response rate of this study is 0%. All subjects agreed to the usage of their sociodemographic characteristics and personality analyses for the study, this study was carried out in accordance to the Declaration of Helsinki and was approved by the local ethics committee, the Domain Specific Review Board (DSRB) of the National Healthcare group, Singapore.

The test consisted of 240 statements, which the respondent agreed or disagreed with. A score was generated for each of the 5 major character traits namely: neuroticism (N), extraversion (E), openness (O), conscientiousness (C), agreeableness (A). Each of these Character traits were subdivided into 6 component facets. According to the inventory, the score of each major character trait and component facets range from 0–100. Raw scores were converted to standardised T-scores using adult norms reported in the Manual and analysed. Univariate analyses of the personality traits were performed using the independent sample T-tests and a significance level of *p* < 0.05 was adopted. Statistical analyses were performed using the Stata IC13.1 (StataCorp, 4905 Lakeway, College Station, TX, 77,845, USA).

## Results

Table 1 shows the sociodemographic characteristics of the residents. There were no differences in demographics in terms of age, race, or training seniority between the medical and surgical residents. Female residents comprised of a higher percentage within the medical residents, accounting for 65.7% of the medical respondents surveyed, compared to 22.9% of the surgical respondents. Medical residents comprised of residents from the subspecialties of general medicine, cardiology, renal medicine, respiratory medicine, rheumatology, infectious disease and geriatrics. The surgical residents who responded came from the division of general surgery, orthopaedics, cardiothoracic, urology, hand surgery, plastic surgery and neurosurgery.

**Table 1:** Sociodemographic characteristics of the residents

Characteristics	Total	Medical	Surgical	*p*-value
Patients, n (%)	70	35 (50.0)	35 (50.0)	
Gender, n (%)				0.0001
Male	39 (55.7)	12 (34.3)	27 (77.1)	
Female	31 (44.3)	23 (65.7)	8 (22.9)	
Age, years				
Mean (SD)	27.9 (2.06)	27.5 (2.05)	28.2 (2.04)	0.165
Median (Interquartile range)	28 (4)	27 (7)	28 (8)	
Race, n (%)				0.602
Chinese	63 (90.0)	32 (91.4)	31 (88.6)	
Indian	6 (8.6)	3 (8.6)	3 (8.6)	
Others	1 (1.4)	0 (0.0)	1 (2.9)	
Training status, n(%)				
Junior residents	58 (82.9)	29 (82.9)	29 (82.9)	
Senior residents	12 (17.1)	6 (17.1)	6 (17.1)	

Independent sample t-tests were performed for 5 traits and their respective facets to derive associations in specialty with personality dimensions, as outlined in Table 2. Medical residents displayed higher scores in the area of overall agreeableness compared to surgical residents, with a mean score of 47.4 and 40.5 respectively (*t* = 2.931, *p* = 0.005). This gives a standardized mean difference (Cohen’s D) of a moderate effect size of 0.71. Within the agreeableness facets, medical residents also displayed higher scores of straightforwardness (*p* = 0.006), compliance (*p* = 0.006) and modesty (*p* = 0.02).

**Table 2:** Analysis of NEOPI scores

NEOPI dimensions	Medical Residents Mean Score	Surgical Residents Mean Score	Size of effect Cohen’s d (**Bold** for significantly different)
N Neuroticism	51.0	54.7	0.39
N1 Anxiety	50.1	51.5	0.14
**N2 Angry Hostility**	**49.8**	**57.1**	**0.66**
N3 Depression	52.5	54.8	0.34
N4 Self-Consciousness	54.4	53.0	0.12
**N5 Impulsiveness**	**47.4**	**54.0**	**0.60**
N6 Vulnerability	47.7	50.1	0.23
**E Extraversion**	**45.4**	**52.4**	**0.56**
E1 Warmth	46.4	46.8	0.03
E2 Gregariousness	47.9	51.2	0.29
**E3 Assertiveness**	**50.2**	**54.6**	**0.51**
E4 Activity	51.1	53.7	0.33
**E5 Excitement Seeking**	**53.9**	**59.1**	**0.57**
E6 Positive Emotions	48.2	51.6	0.29
O Openness to Experience	52.6	49.4	0.32
O1 Openness to Fantasy	53.5	51.9	0.15
O2 Openness to Aesthetics	50.6	49.2	0.15
O3 Openness to Feelings	47.9	50.8	0.3
O4 Openness to Actions	48.1	47.2	0.09
O5 Openness to Ideas	54.2	51.2	0.29
O6 Openness to Values	49.5	46.9	0.29
**A Agreeableness**	**47.7**	**40.5**	**0.71**
A1 Trust	44.7	41.5	0.26
**A2 Straightforwardness**	**49.1**	**41.1**	**0.68**
A3 Altruism	47.4	46.4	0.09
**A4 Compliance**	**49.0**	**40.5**	**0.69**
**A5 Modesty**	**51.3**	**45.4**	**0.58**
A6 Tender-Mindedness	48.8	47.7	0.10
C Conscientiousness	50.1	51.5	0.14
C1 Competence	47.2	49.1	0.19
C2 Order	45.6	47.8	0.20
C3 Dutifulness	50.4	50.7	0.03
C4 Achievement Striving	53.5	54.9	0.12
C5 Self-Discipline	44.8	44.1	0.07
C6 Deliberation	53.5	49.7	0.40

Surgical residents displayed higher scores in terms of overall extraversion, with a mean of 52.4 against a mean of 45.4 for medical residents (*t* = -2.322, *p* = 0.023), which corresponded to a moderate effect size. Within the extraversion facets, surgical residents were also statistically more assertive (*p* = 0.041) and excitement-seeking (*p* = 0.02).

There was no difference in the overall neuroticism domain between medical and surgical specialties, however, within the neuroticism facets, surgical residents had statistically higher mean scores in angry hostility and impulsiveness. Medical and surgical residents also did not differ significantly on the character traits of openness or conscientiousness. Gender stratification did not result in any changes in the results which were statistically significant.

Within the surgical residents, urology residents which comprised 31.4% of the surgical residents, displayed statistically significant lower scores in the area of achievement striving compared to non-urology residents (mean of 49 vs 57, *p* = 0.048). They also had higher scores for the trait of agreeableness to non-urology surgical residents (45 vs 38, *p* = 0.024).

## Discussion

In this study we compared the personality profiles of a sample of medical residents with a sample of surgical residents from NUHS. Results suggest that fundamental differences exist between the two groups of residents. Congruent with previous studies [10–13], Our results showed that medical residents scored higher in ‘agreeableness’, people who are more agreeable tend to be more cooperative, kind, sympathetic as oppose to being harsh or rude. These qualities are unsurprising, given the intellectual rigors in the realm of internal medicine, where open discussion and sharing of ideas are integral to the success of a medical team. This is in contrast to the surgical residents, where individual and absolute decision-making skills are often essential to a successful surgery, as compared to their ability to work well with others. Surgical residents, on the other hand, scored higher for ‘extraversion’, which points towards someone who is more enthusiastic, verbal, assertive as opposed to being withdrawn, reserved or shy. Within the extroversion facet, surgical residents displayed greater assertiveness and excitement-seeking behavior. This is also a consistent finding in previous studies [4, 12, 13], and may be accounted by the more rapid and action-oriented nature of a surgical job.

Having deeper understanding of the personality difference between different groups of residents can be beneficial is several ways. Firstly, medical students and junior doctors may take the same personality tests to gain better insight into their own personalities, and thereafter compare the results to current residents. This may help make a more informed decision when choosing specialties. They may find it more enriching to be surrounded by like-minded colleagues and gain a stronger support system among colleagues with similar personalities.

Secondly, understanding personalities of successful residents can be beneficial to more junior residents. Periodic personality assessments can be done throughout a resident’s journey through residency and such tests could help to highlight traits that can potentially be detrimental to work performance. In doing so, it can allow oneself to be more mindful and work on these traits. In addition, mentors may be able to provide personalized advice on how to work on these traits to improve work performance. Success in this form of training have been seen in other sectors such as military pilots [14], whereby it has been shown that personalities can be altered and in doing so, likelihood of a safe mission can be improved. In the similar study, there were also evidence to suggest that different types of training can benefit trainees of a certain personality.

It is believed that burnout and personality traits are closely related [15, 16], these assessments can also help residency programs to identity residents who are potentially suffering from burnout early so that early intervention can be provided. Ultimately, this might help to lower the residency attrition rate, hence minimizing the social and economic impacts that comes along with it.

In the context of Singapore, junior doctors may choose to undergo these personality tests during their PGY1 year to gain more insight into their own personality traits before their residency application. This need not be compulsory nor part of a formal assessment for residency. With their newly gained insight, they may then go on to make better career decisions. By choosing the most fitting residency, junior doctors may be better positioned to use their innate strengths and perform better at work. This is can also increase work satisfaction and reduce burn out.

Our study did not take into account the motivation and success of sample of medical residents and surgical residents. Hence, the results of this study may not be representative of residents who are ultimately successful at the end of their training. Personalities are known to be affected by age, longitudinal studies in general population have shown that people tend to become more agreeable, conscientious while being less neurotic, extroverted and open as they age [17], personality traits might also evolve as one goes through residency [18]. However, despite this limitation, our study was still able to report similar findings between our study and other studies performed on board-certified physicians [4, 12, 13]. Secondly, our study is also inherently limited by demand characteristic bias due to its self-assessment nature, differences may exist between self-assessment and external assessment of personality and the concerns of faking responses have also been previously raised for studies involving personality tests [19, 20]. Although the NEO-PI-R questionnaire have questions to mitigate this factor, specialized questionnaires such as the Balanced Inventory of Desirable Responding (BIDR) [21] would have been more ideal. In our case, we have decided to omit using such toolkits to minimize responder fatigue. Thirdly, our study is a cross-sectional study performed in a single institution. Hence, the results may not apply general to all settings and across different time points of a physician’s career, further prospective studies with larger numbers are needed to confirm the associations found in our study. Longitudinal studies looking at not only the differences between personalities of surgical and medical residents, but also how they change as they go through residency will be greatly beneficial as well; however, such studies are still lacking in current literature.

## Conclusion

This study suggests that there are fundamental differences between the personalities of medical and surgical residents.​ Detailed analysis of each individual’s data could be useful, with proper assistance and coaching, for residents in learning more about their personalities and how these traits serve to impact their clinical practice. This can be beneficial in future career counseling and the development of a more holistic medical practitioner. Moving into the future, standardized and validated personality profile testing such as the Revised NEO Personality inventory can become a standard practice in the longitudinal follow up of a resident to aid resident evaluation, mentoring and career development.

## Data Availability

The datasets used and/or analysed during the current study available from the corresponding author on reasonable request.

## Abbreviations

NEO PI-R: Revised NEO Personality Inventory
NUHS: National University Hospital System
DSRB: Domain Specific Review Board

## Footnotes

1 Inventory can be obtained at https://www.parinc.com/Products/Pkey/276
